# 4′-*O*-methylhonokiol increases levels of 2-arachidonoyl glycerol in mouse brain via selective inhibition of its COX-2-mediated oxygenation

**DOI:** 10.1186/s12974-015-0307-7

**Published:** 2015-05-13

**Authors:** Andrea Chicca, Maria Salomé Gachet, Vanessa Petrucci, Wolfgang Schuehly, Roch-Philippe Charles, Jürg Gertsch

**Affiliations:** Institute of Biochemistry and Molecular Medicine, NCCR TransCure, University of Bern, Bühlstrasse 28, CH-3012 Bern, Switzerland; Institute of Zoology, Karl-Franzens-University Graz, Universitätsplatz 2, 8010 Graz, Austria

**Keywords:** 4′-*O*-methylhonokiol, Endocannabinoids, COX-2, CB2 receptor, Polypharmacology, Partial agonist, Endocannabinoid system, 2-arachidonoyl glycerol, Magnolia grandiflora

## Abstract

**Background and purpose:**

4′-*O*-methylhonokiol (MH) is a natural product showing anti-inflammatory, anti-osteoclastogenic, and neuroprotective effects. MH was reported to modulate cannabinoid CB2 receptors as an inverse agonist for cAMP production and an agonist for intracellular [Ca2+]. It was recently shown that MH inhibits cAMP formation via CB2 receptors. In this study, the exact modulation of MH on CB2 receptor activity was elucidated and its endocannabinoid substrate-specific inhibition (SSI) of cyclooxygenase-2 (COX-2) and CNS bioavailability are described for the first time.

**Methods:**

CB2 receptor modulation ([35S]GTPγS, cAMP, and *β*-arrestin) by MH was measured in *h*CB2-transfected CHO-K1 cells and native conditions (HL60 cells and mouse spleen). The COX-2 SSI was investigated in RAW264.7 cells and in Swiss albino mice by targeted metabolomics using LC-MS/MS.

**Results:**

MH is a CB2 receptor agonist and a potent COX-2 SSI. It induced partial agonism in both the [35S]GTPγS binding and *β*-arrestin recruitment assays while being a full agonist in the cAMP pathway. MH selectively inhibited PGE2 glycerol ester formation (over PGE2) in RAW264.7 cells and significantly increased the levels of 2-AG in mouse brain in a dose-dependent manner (3 to 20 mg kg^−1^) without affecting other metabolites. After 7 h from intraperitoneal (i.p.) injection, MH was quantified in significant amounts in the brain (corresponding to 200 to 300 nM).

**Conclusions:**

LC-MS/MS quantification shows that MH is bioavailable to the brain and under condition of inflammation exerts significant indirect effects on 2-AG levels. The biphenyl scaffold might serve as valuable source of dual CB2 receptor modulators and COX-2 SSIs as demonstrated by additional MH analogs that show similar effects. The combination of CB2 agonism and COX-2 SSI offers a yet unexplored polypharmacology with expected synergistic effects in neuroinflammatory diseases, thus providing a rationale for the diverse neuroprotective effects reported for MH in animal models.

**Electronic supplementary material:**

The online version of this article (doi:10.1186/s12974-015-0307-7) contains supplementary material, which is available to authorized users.

## Introduction

4′-*O*-methylhokiol (MH) is the major bioactive constituent of *Magnolia grandiflora* L. seed oil and shows pronounced anti-inflammatory, anti-osteoclastogenic, and neuroprotective effects [1-4]. In mouse models, MH prevents LPS-induced memory loss and *β*-amyloid (1 to42) accumulation [2]. Moreover, MH appears to efficiently attenuate the development of Alzheimer’s disease in Tg2576 transgenic mice [3]. The proposed underlying mechanisms appear to be linked to the inhibition of nuclear factor kappa B, the gene expression of inducible nitric oxide synthase and cyclooxygenase-2 (COX-2), as well as cannabinoid type-2 (CB_2_) receptors [2,5]. MH and some derivatives potently inhibit COX-2 with poor selectivity towards the constitutive isoform COX-1 [6,7]. Furthermore, MH attenuates different signaling cascades related to oxidative stress and MAP kinases, inhibits moderately acetylcholinesterase activity (see [5] for review), and it also acts as positive allosteric GABA_A_ receptor ligand [8]. Schuehly *et al.* [1] for the first time described the relatively potent and selective CB_2_ receptor binding of MH and different analogs. MH was shown to exert an unusual dualistic modulation of CB_2_ receptor activity, acting as an inverse agonist at the cAMP pathway while behaving as an agonist at increasing the intracellular levels of [Ca^2+^] [1]. The CB_2_ receptor inverse agonism on the cAMP pathway was described as the relevant mechanism associated to anti-osteoclastogenesis effects described in mouse RAW264.7 cells and primary human monocytes/macrophages. Other authors recently reported that MH behaves as a CB_2_ receptor agonist in the cAMP pathway [9], pointing out discrepancies in receptor assays, which gave impetus to the present in-depth study on the CB_2_ receptor pharmacology of MH. The main degradation route of endocannabinoids (ECs) is their hydrolysis by fatty acid amide hydrolase (FAAH) (for anandamide (AEA)) and monoacylglycerol lipase/α, β-hydrolases (MAGL/ABHDs) (for 2-arachidonoyl glycerol (2-AG)), but ECs might undergo additional enzymatic modifications including oxygenation mediated by COX-2, lipoxygenases (LOXs), and cytochrome P450 (CYP450) [10-12]. Several studies emphasized the relevance of COX-2-mediated oxygenation of ECs *in vitro* and *in vivo* under specific conditions like inflammation and in FAAH knock-out animals [13-15]. EC oxygenation might be particularly relevant in inflammation or tissues that constitutively express the inducible form of COX such as the brain, kidney, and spinal cord [16-18]. Upon COX-2 activity, AEA and 2-AG are converted into prostaglandin ethanolamides (prostamides) and prostaglandin glycerol esters (PG-GEs), respectively [10,11]. Prostamides and PG-GEs exert pro-inflammatory actions probably by interacting with specific targets that are different from cannabinoid and classic prostaglandin receptors. A heterodimeric association between the wild-type prostaglandin F (FP) receptor and an alternative splicing variant (Alt4) of such receptors was identified as molecular target for prostamideF_2α_ [19]. The hyperalgesic actions of prostamideF_2α_ were then confirmed by using selective receptor antagonists which showed antinociceptive effects *in vitro* and *in vivo* [15,20]. The inhibition of prostamide and PG-GEs formation by COX-2 substrate-specific inhibitors (SSIs) has been reported. Intriguingly, such inhibitors can lead to an increase of AEA and 2-AG levels without affecting the levels of arachidonic acid (AA) and PGE_2_ [14,21,22]. Duggan *et al.* [14] recently showed that (*R*)-flurbiprofen acts as COX-2 SSI by increasing EC levels and by reducing prostamide formation without affecting AA and PGE_2_ levels. In addition, the morpholino amide derivative of indomethacin LM-4131 showed potent anxiolytic effects in several animal models by selectively inhibiting EC oxygenation, increasing levels of AEA and 2-AG in the brain [21]. COX-2 SSI might represent a promising anti-inflammatory strategy to circumvent the typical gastrointestinal and cardiovascular aversive effects of the non-selective COX-2 inhibitors.

Here, we show that MH and some derivatives exert dual and potentially synergistic actions on the endocannabinoid system (ECS) by acting as central nervous system (CNS) penetrating SSIs of COX-2 and as CB_2_ receptor agonists. We provide detailed analyses on CB_2_ receptor signaling and COX-2-mediated oxygenation of ECs by using purified *h*COX-2, RAW264.7 cells, and LPS-challenged mouse brain.

## Methods

### Materials

[Ethanolamine-1-^3^H]-anandamide and [1,2,3-^3^H]-2-arachidonyl glycerol were obtained from American Radiolabeled Chemicals (St. Louis, MO, USA), while the [^3^H] CP-55,940 was obtained from PerkinElmer Life Sciences (Waltham, MA, USA). AM630, WIN55-212, AEA, 2-AG, DuP-697, arachidonic acid-*d8* (5Z,8Z,11Z,14Z-eicosatetraenoic-5,6,8,9,11,12,14,15-*d8* acid), AEA-*d4* (N-(2-hydroxyethyl-1,1,2,2-*d4*)-5Z,8Z,11Z,14Z-eicosatetraenamide) 2-AG-*d5* (5Z,8Z,11Z,14Z-eicosatetraenoic acid, 2-glyceryl-1,1,2,3,3-*d5* ester), LEA-*d4* (N-(2-hydroxyethyl-1,1,2,2-*d4*)-9Z,12Z-octadecadienamide); OEA-*d4* (N-(2-hydroxyethyl-1,1,2,2-*d4*)-9Z-octadecenamide), PEA-*d5* (N-(2-hydroxyethyl)-hexadecanamide-15,15,16,16,16-*d5*); PGE_2_-*d4* (9-oxo-11α,15S-dihydroxy-prosta-5Z,13E-dien-1-oic-3,3,4,4-*d4* acid); and PGE_2_-1-glyceryl ester-*d5* (9-oxo-11α,15S-dihydroxy-prosta-5Z,13E-dien-1-oic acid,1-glyceryl ester-*d5*) were obtained from Cayman Chemicals Europe (Ann Arbor, MI, USA). MH-*d6* (2-(4-methoxy-3-prop-2-enylphenyl (*d*))-4-prop-2-enylphenol (*d*)). The structures of the MH derivatives were previously published in Schühly *et al.* [7] and Schuehly *et al.* [1]. The procedure of isolation and purification of MH and the synthesis of its derivatives was previously reported in [1,7]. All tubes used for assays (plastic and glass) were silanized.

### Animals

All study involving animals are reported in accordance with the Swiss Federal guidelines. Female RjOrl: SWISS (60) 7 to 8 weeks old, provided by Janvier Labs (St Berthevin, France), were used for these experiments. The mice were housed in groups of five per cage in a selected pathogen-free unit under controlled 12-h light/12-h dark cycle, ambient temperature 21°C ± 1°C humidity 40% to 50% with free access to standard rodent chow and water. The mice were acclimatized to the Animal House for at least 3 days before the experiment.

### Radioligand displacement assays on hCB_1_ and hCB_2_ receptors

Receptor binding experiments were performed with membrane preparations as previously reported [23]. Briefly, clean membranes expressing *h*CB_1_ or *h*CB_2_ were resuspended in binding buffer (50 mM Tris-HCl, 2.5 mM EDTA, 5 mM MgCl_2_, 0.5 mg mL^−1^ fatty acid-free bovine serum albumin (BSA), pH 7.4) and incubated with vehicle or compounds and 0.5 nM of [^3^H] CP55,940 for 2 h at 30°C. Non-specific binding was determined in the presence of 10 μM of WIN55,512. After incubation, membranes were filtered through a pre-soaked 96-well microplate bonded with GF/B filters under vacuum and washed twelve times with 150 μL of ice-cold binding buffer. The radioactivity was measured and the results expressed as [^3^H] CP55,940 binding.

### [^35^S]GTPyS assay

Assays were performed as previously described [24]. Briefly, 5 μg of clean membranes expressing *h*CB2 were diluted in binding buffer (50 mM Tris-HCl, 3 mM MgCl_2_, 0.2 mM EGTA, and 100 mM NaCl at pH 7.4 plus 0.5% fatty acid-free BSA) in the presence of 10 μM of GDP and 0.1 nM of [^35^S]GTPγS. The mixture was kept on ice until the binding reaction was started by adding the vehicle or compounds. Non-specific binding was measured in the presence of 10 μM of GTPγS. The tubes were incubated at 30°C for 90 min. The reaction was stopped by rapid filtration through a 96-well microplate bonded with GF/B filters previously pre-soaked with washing buffer (50 mM of Tris-HCl pH 7.4 plus 0.1% fatty acid-free BSA). The filters were washed six times with 180 μL of washing buffer under vacuum. Experiments were performed in the presence and after removal of constitutive activity of CB_2_ receptors. In the latter, membranes were obtained from cells treated overnight with 10 μM of AM630 to shift the vast majority of the receptor population in the inactive state. After the incubation, cells were washed six times with 10 mL of culture medium for 10 min each. Finally, cells were detached and membranes prepared and used to perform [^35^S]GTPγS binding assays. Membrane obtained from mock-transfected CHO cells were used as negative control. Membranes derived from mouse spleen (20 μg) and human promyelocytic leukemia HL60 cells (40 μg) were used as sources of CB_2_ receptors expressed in native conditions. In order to reduce the basal [^35^S]GTPγS binding, the experiments were performed after 30-min pre-treatment with adenosine deaminase (5 units mL^−1^). The radioactivity was measured, and the results were expressed as [^35^S]GTPγS binding.

### cAMP assay

cAMP assays were performed in CHO-*h*CB2 stably transfected with the pGloSensorTM 22-F plasmid (Promega, Madison, WI, USA) as previously described [22]. Briefly, cells were seeded in 96-well plate and kept in an equilibration medium (GloSensor™ cAMP reagent diluted in 5% of a CO_2_-independent medium plus 10% FBS) for 2 h at RT in the dark. Compounds or vehicle were diluted in an assay medium containing 1 μM of forskolin and 250 μM of 3-isobutyl-1-methylxanthine (IBMX). Chemiluminescence was recorded after 20 min. Non-specific signal was determined in cells with only IBMX. Experiments were performed in the presence and after removal of the constitutive activity of the receptors following the same procedure described above. Results were normalized by subtracting the residual cAMP production and expressed amount of cAMP formed.

### β-arrestin assay

The PathHunter® β-arrestin cells (CHO-K1-HOMSA-CNR2) were gently donated by DiscoverX (via Hoffman La-Roche Ltd, Basel, Switzerland) and cultured in an F-12 medium containing 10% FBS, 200 μg mL^−1^ hygromycin and 800 μg mL^−1^ geneticin. For the experiments, 2 × 10^4^ cells were seeded in 96-well plate and left overnight in the incubator. The day after, vehicle or compounds were diluted in 40 μL of a CO_2_-independent medium plus 10% FBS. The mixture was added to the cells and incubated for 90 min at 37°C. Chemiluminescence was detected using the PathHunter® detection system (DiscoveRx Corporation, Fremont, CA, USA) according to the instruction protocol. Background levels were subtracted and the results expressed as β-arrestin recruitment.

### COX-2 inhibition assay *in vitro*

The experiments were performed as previously described [22]. Briefly, the inhibition of recombinant *h*COX-2 was assessed using a COX fluorescent inhibitor screening assay kit from Cayman Chemicals Europe. Tested compounds or vehicle were pre-incubated with COX-FIS assay buffer (Tris-HCl 100 mM, pH 8), 1 μM of COX-FIS heme *h*COX-2 FIS assay agent, and 30 μM of ADHP (10-acetyl-3,7-dihydroxyphenoxazine) fluorometric substrate for 15 min at RT. The reaction was started by adding arachidonic acid, AEA, or 2-AG (10 μM). Fluorescence intensity was measured (535/580 nm) after 5 min of incubation. The results were expressed as *h*COX-2 activity.

### LC-MS/MS quantification of ECs and PGs in RAW264.7 cells

In case of experiments with homogenates, cells were seeded in T75-cm^2^ flask and stimulated with LPS (1 μg mL^−1^) for 12 h at 37°C. After incubation, cells were homogenized in assay buffer (Tris-HCl 10 mM, pH 8) and 900 μg of total protein were used to perform the COX-2 assay. When using intact cells, 0.5 × 10^6^ cells were seeded in a 24-well plate and challenged with LPS (1 μg mL^−1^) for 12 h at 37°C. The medium was discarded and replaced by a medium containing vehicle or compounds. After 30 min of incubation at 37°C, 2-AG (10 μM) was added and plates were incubated for an additional 30 min at 37°C. Further experiments were carried out without adding 2-AG but stimulating its cellular biosynthesis. In this setting, RAW264.7 cells were treated for 8 h with LPS (1 μg mL^−1^), ATP (1 mM), and thapsigargin (2 μM) in the presence of vehicle or compounds. In both experimental conditions, after the incubation, cells were detached, transferred into a chloroform:methanol mixture (2:1) containing the internal standards (ISs), and then, phosphate buffered saline (PBS) was added to the final ratio of 6:3:1.5 (chloroform:methanol:PBS). The suspension was vortexed, sonicated, and centrifuged for 5 min at 800 g at 4°C. The organic phase was recovered and dried under nitrogen. The quantification of AA, 2-AG, PGE_2_, and PGE_2_GE was performed using LC-MS/MS. The multiple reaction monitoring (MRM) parameters (precursor ion/product ion, declustering potential, collision energy) used in the survey and the respective IS are detailed in Gachet *et al.* [25] for all analytes except for: MH (Q): 279/264 m/z, −70 eV, −23 eV and (q): 279/248 m/z, −70 eV, −40 eV; MH-*d*6: 285/279 m/z, −82 eV, −24 eV; PGE_2_GE (Q): 444/409 m/z, 40 eV, 13 eV and (q): 444/373 m/z, 40 eV, 19 eV; and PGE_2_GE-*d5*: 449/396 m/z, 43 eV, 17 eV. The quantification was based on the area ratio of analytical standard/IS.

### LC-MS/MS quantification of ECs and PGs *in vivo*

Swiss albino mice (females, 8 weeks old) were injected intraperitoneally (i.p.) with vehicle or MH at different doses (3, 10, and 20 mg kg^−1^). Female mice were used for ethical reasons because they were less susceptible for neuroinflammatory damage in this model. After 1 h, the animals (6 to 14 animals per group) were injected i.p. with LPS (2.5 mg kg^−1^) and kept in the dark for an additional 6 h. After anesthetization, mice were perfused with PBS and the brains were collected. Lipid extraction was performed as recently described (Gachet *et al.* [25]). In brief, half brain was homogenized on a BeadBeater (Mini-BeadBeater-24, BioSpec, Bartlesville, OK USA) using Chrome-Steel Beats (2.3 mm dia., BioSpec) in the presence of ice-cold chloroform:methanol (2:1) for 1 min (3,450 strokes min^−1^) at 4°C. Homogenates were added with PBS (containing ISs) to the final ratio of 6:3:1.5 (chloroform:methanol:PBS). The suspension was vortexed, sonicated, and centrifuged for 5 min at 800 g at 4°C. The organic phase was recovered and dried under nitrogen. Subsequently, the samples were reconstituted in ethanol and after the addition of water and pH adjustment (pH = 3), an aliquot was extracted using C-18Sep-Pak cartridge (Waters AG, Zug, Switzerland). Cartridges were washed with 10% ethanol and eluted with acetonitrile (ACN)/ethyl acetate (1:1). The eluates were collected and evaporated to dryness under nitrogen. The samples were reconstituted in ACN, centrifuged for 5 min at 16,100 g at 4°C, and measured by LC-MS/MS in negative and positive modes. The quantification was based on the area ratio of analytical standard/IS.

### [^3^H]AEA and [^3^H]2-AG hydrolysis assay

The experiments were performed as previously described [23]. Briefly, vehicle or compounds were pre-incubated for 30 min at 37°C with 200 μg of pig brain homogenate for FAAH and MAGL and 100 μg of BV-2 cell homogenate for ABHDs in assay buffer (10 mM Tris-HCl, 1 mM EDTA, pH 7.6 plus 0.1% fatty acid-free BSA). To measure FAAH activity, a mixture of AEA/[^3^H] AEA was added to the homogenate (final concentration of 1 μM) and incubated for 15 min at 37°C. After incubation, tubes were added with chloroform:methanol (1:1), vortexed, and centrifuged at 10,000 rpm for 10 min at 4°C. To measure MAGL/ABHDs activity, AEA and [^3^H]AEA were replaced by 2-oleoylglycerol (2-OG) and [^3^H]2-OG, respectively. The radioactivity associated to the [^3^H]ethanolamine (or [^3^H]glycerol) formation was measured in the aqueous phase. The results were expressed as enzymatic activity.

### [^3^H]AEA uptake into U937 cells

The experiments were performed as previously described [23]. Briefly, 10^6^ of U937 cells were suspended in 0.5 mL of PBS and pre-incubated with vehicle or compounds for 30 min at 37°C. Successively, cells were added with a mixture of AEA/[^3^H]AEA (final concentration of 100 nM) and incubated for 5 min at 37°C. The uptake process was stopped by rapid centrifugation at 800 g for 5 min at 4°C. The supernatant was discarded and the pellet washed with ice-cold PBS plus 1% of fatty acid-free BSA. Cell pellets were resuspended in PBS and extracted with chloroform:methanol (1:1). The organic phase was collected and the radioactivity measured. The results were expressed as [^3^H]AEA uptake. In case of U937 cell-derived macrophages, U937 cells were treated for 48 h with 2 ng mL^−1^ of phorbol 12-myristate 13-acetate (PMA) and the uptake experiments were performed as described above.

### Data analysis

Data were collected from at least three independent experiments each performed in triplicate. Results are expressed as mean values and standard deviation (SD). The [^35^S]GTPγS assays performed using CHO-hCB_2_ membranes were repeated five times, and the error was expressed as standard error of the mean (SEM). The statistical significance difference among groups was determined by Student’s *t* test (paired, two-tailed *t* test) or one-way ANOVA followed by Bonferroni’s post-test. Statistical differences between treated and control groups were considered as significant if *P* ≤ 0.05. GraphPad 5.0 software was used to fit the concentration-dependent curves and for the statistical analysis.

## Results

**Figure 1:**
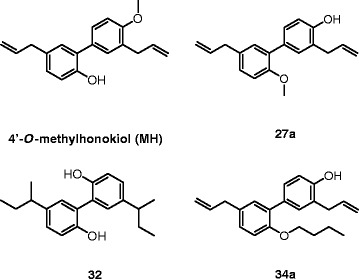
Chemical structures of 4′-*O*-methylhonokiol (-4′-methoxy-5,3′-di-(2-propenyl)-biphenyl-2-ol), **27a** (2-methoxy-5,3′-di-(2-propenyl)- biphenyl-4′-ol), **32** (5,5′-di-sec-butyl- biphenyl-2,2′-diol) and **34a** (2-butyloxy-5,3′-di-(2-propenyl)- biphenyl-4′-ol).

**Figure 2:**
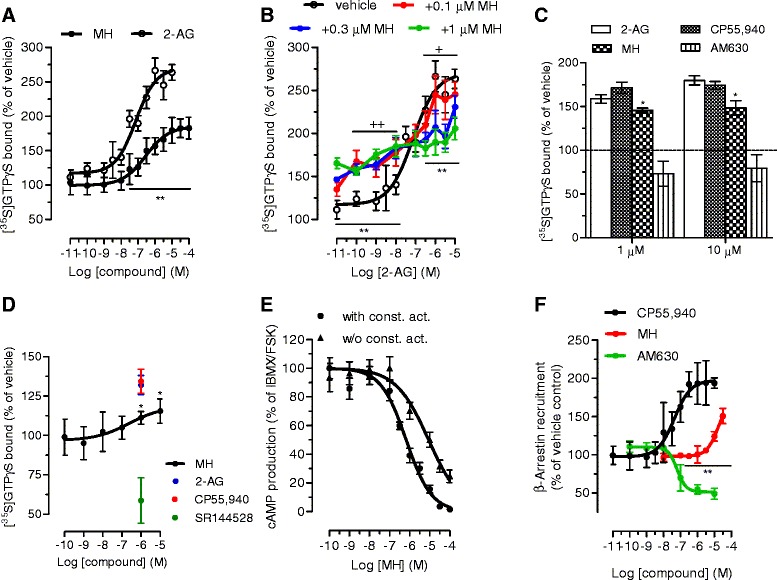
Modulation of CB_2_ receptor activity by MH. **(A)** [^35^S]GTPγS binding assay was performed in stably *h*CB_2_ receptor overexpressing CHO-K1 membranes, in the presence of different concentrations of MH and 2-AG alone or **(B)** in combination. The same experiments were performed in endogenously CB_2_ expressing **(C)** HL60 cells and **(D)** mouse spleen membranes. **(E)** Inhibition of forskolin-induced cAMP formation by MH in CHO-*h*CB_2_ cells transfected with pGloSensorTM 22-F plasmid. The experiments were performed in the presence (circles) and in the absence (triangles) of constitutive activity. **(F)** *β*-arrestin recruitment induced by increasing concentrations of MH, CP55,940, and AM630, measured in PathHunter® *β*-arrestin cells (CHO-K1-HOMSA-CNR2). ^**^ *P* < 0.01 MH *vs.* 2-AG (Figure 2A); ^**^ *P* < 0.01 MH (1 μM) plus 2-AG *vs.* 2-AG, ^++^ *P* < 0.01, ^+^ *P* < 0.05 MH (0.3 μM) plus 2-AG *vs.* 2-AG (Figure 2B). ^*^ *P* < 0.05 MH *vs.* 2-AG and CP55,940 (Figures 2C, D).^**^ *P* < 0.01 MH *vs.* CP55,940 (Figure 2F).

**Figure 3:**
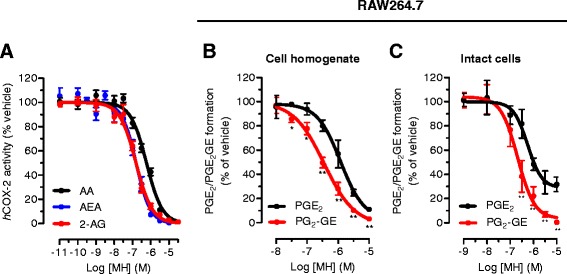
Dose-dependent COX-2 inhibition induced by MH in different biological matrices. **(A)** MH-mediated inhibition of *h*COX-2 using 10 μM of AA (black), AEA (blue), and 2-AG (red) as substrates. MH-mediated inhibition of PGE_2_ and PGE_2_-GE formation in **(B)** RAW264.7 cell homogenate and **(C)** intact cells after 12 h of incubation with LPS (1 mg mL^−1^). Homogenates and intact cells were incubated for 30 min with 10 μM of 2-AG after the pre-treatment (30 min) with different concentrations of MH or vehicle. ^*^ *P* < 0.05, ^**^ *P* < 0.01 PGE_2_ *vs.* PGE_2_-GE.

**Figure 4:**
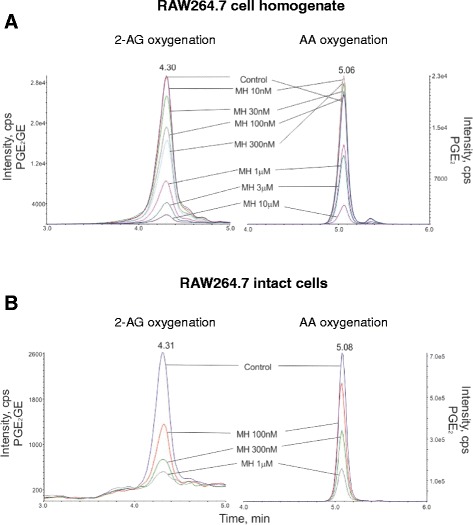
MS chromatograms of PGE_2_ and PGE_2_-GE measured in different biological matrices. Quantification of PGE_2_ and PGE_2_-GE formation measured after incubation of **(A)** RAW264.7 cell homogenate and **(B)** intact cells with 10 μM of AA or 2-AG in the presence of different concentrations of MH or vehicle. Homogenates and cells were treated for 12 h with LPS (1 mg mL^−1^) followed by 30-min incubation with 10 μM of 2-AG after the pre-treatment (30 min) with different concentrations of MH or vehicle.

**Figure 5:**
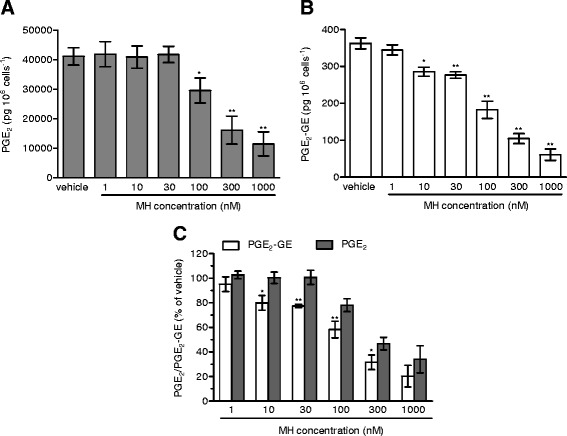
MH-mediated inhibition of PGE_2_ and PGE_2_-GE formation in RAW264.7 cells without external addition of 2-AG. Concentration-dependent inhibition of **(A)** PGE_2_ and **(B)** PGE_2_-GE formation (picogram per million of cell) in RAW264.7 intact cells previously stimulated with LPS (1 mg mL^−1^) ATP (1 mM) and thapsigargin (2 μM) for 8 h. **(C)** PGE_2_ and PGE_2_-GE formation inhibition upon different concentrations of MH (expressed as % of vehicle-treated cells). ^*^ *P* < 0.05, ^**^ *P* < 0.01 MH treated samples *vs.* vehicle for panels (A) and (B); ^*^ *P* < 0.05, ^**^ *P* < 0.01 PGE_2_-GE *vs.* PGE_2_ for panel (C).

**Figure 6:**
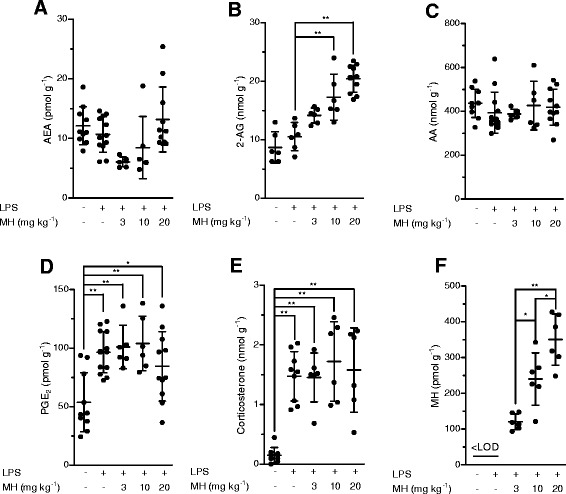
LC-MS/MS quantification of different analytes in mouse brain. **(A)** AEA, **(B)** 2-AG, **(C)** AA, **(D)** PGE_2_, and **(E)** corticosterone were quantified in the brains from mice (6 to 14 animals per group) challenged for 6 h with LPS (i.p., 2.5 mg kg^−1^, or saline), after 1 h of pre-treatment with MH (i.p., 3, 10, and 20 mg kg^−1^) or vehicle. **(F)** MH quantification in the brain of LPS-challenged mice. ^*^ *P* < 0.05, ^**^ *P* < 0.01 treated *vs.* not-treated animals (Figure 6A-E) and ^*^ *P* < 0.05 MH 10 mg kg^−1^ *vs.* 20 mg kg^−1^ (Figure 6F).

First, we confirmed that MH (Figure 1) selectively binds to CB_2_ receptors with a *K*_i_ value of 188.5 (131.7 to 269.4) nM over 2.4 (1.9 to 2.9) μM for CB_1_ receptors (Table 1). MH induced a concentration-dependent increase of [^35^S]GTPγS binding with an EC_50_ value of 285.7 (125.6 to 647.2) nM and a maximal binding of 185.7% (167.1 to 204.3) (Figure 2A). In the same assay, the positive control 2-AG-induced [^35^S]GTPγS binding with an EC_50_ value of 74.0 (28.4 to 192.9) nM, reaching 252.8% (210.2 to 295.4) of maximal response. Statistical analyses confirmed that MH induced a lower maximal G-protein recruitment compared to the endogenous full agonist 2-AG, thus suggesting partial agonism. Partial agonists are versatile ligands which can behave as agonists or antagonists depending on the conditions [26]. In Figure 2B, the incubation of fixed concentrations of MH with increasing concentrations of 2-AG exemplifies these opposite pharmacological behaviors. While MH induced G-protein recruitment at inactive 2-AG concentrations, it reduced the effects induced by high concentrations of the full agonist. We also investigated the behavior of MH using human promyelocytic leukemia HL60 cells [27] and mouse spleen [28] which both endogenously express CB_2_ receptors. As reported in Figure 2C,D, 2-AG, CP55,940, and MH behaved as agonists by increasing the [^35^S]GTPγS binding compared to vehicle, while SR144528 and AM630 reduced G-protein recruitment, thus behaving as inverse agonists. In agreement with the results obtained in CHO-*h*CB_2_ membranes, MH induced a sub-maximal binding of [^35^S]GTPγS compared with the full agonists CP55,940 and 2-AG, in both HL60- and spleen-derived membranes, thus confirming its partial agonist behavior. In addition, we also tested 2-AG, CP55,940, and MH effects using membranes derived from mock-transfected CHO cells. All ligands showed no change of the basal level of [^35^S]GTPγS binding (Additional file 1: Figure S1). Altogether, these data indicate that MH behaves as an agonist at CB_2_ receptors in both overexpressing and native conditions. We next characterized the effects of MH on the CB_2_ receptor-mediated inhibition of forskolin-induced cAMP production. As shown in Figure 2E, increasing concentrations of MH inhibited cAMP formation with an IC_50_ value of 674 (481 to 944) nM. In this assay, in contrast to previous results [1], MH behaved as a full agonist producing the same maximal inhibition as the full agonist 2-AG (data not shown). CB_2_ receptors are known to possess a high intrinsic constitutive activity. Therefore, compounds that act as ‘protean agonists’ can behave differently depending on the proportion of constitutively activated receptors. ‘Protean agonists’ bind to the receptor and stabilize an active conformation that is capable of initiating signal where there is none (inactive state of the receptor), but they trigger an active state of the receptor that is less efficacious than the naturally occurring, spontaneously formed constitutive active state [29]. Thus, ‘protean agonists’ can behave as an agonist or inverse agonist depending on the active state of the receptor. This phenomenon has been accurately investigated over the past few years, and it was found that by pre-treating CB_2_ receptors overnight with the inverse agonist AM630, most of the receptors shift towards the inactive state, thus abolishing the constitutive activity [30]. For example, an inverse agonist becomes a silent antagonist [30] and a protean agonist can switch from an inverse agonist to an agonist or antagonist [30,31]. We have validated our assay system by showing that after removing the constitutive activity, AM630 and SR144528 switched from an inverse agonist to a silent antagonist, while CP55,940 did not change its behavior (Additional file 1: Figure S2) in agreement with the literature [30,32]. As shown in Figure 2E, MH acted as a full agonist in both conditions, suggesting that in our assay, the level of constitutive activity of CB_2_ receptors did not qualitatively affect the pharmacological behavior of MH. We also investigated the effect of MH on *β-*arrestin recruitment. In Figure 2F, it is clearly shown that similarly to [^35^S]GTPγS results, the compound induced a sub-maximal effect compared to CP55,940, therefore behaving as a partial agonist. MH has been shown to inhibit the COX-2-mediated AA oxygenation [7]. However, COX-2 is responsible also for the oxygenation of AEA and 2-AG leading to the formation of PG-EAs and PG-GEs, respectively [10,14,21,33]. Therefore, we next studied whether MH selectively inhibits the COX-2-mediated oxygenation of ECs. In Figure 3A, the concentration-dependent inhibition of purified *h*COX-2 is shown using 10 μM of AEA, 2-AG, and AA as substrates. Noteworthy, MH inhibited the oxygenation of ECs with IC_50_ values of 158 (123 to 204) nM and 156 (129 to 189) nM for AEA and 2-AG, respectively, while it blocked AA oxygenation with an IC_50_ value of 588 (499 to 693) nM (Table 1). We then confirmed the apparent SSI of COX-2 in LPS-stimulated RAW264.7 cells by using cell homogenates and intact cells. We quantified the amount of PGE_2_ and PGE_2_-GE formation by LC-MS/MS analysis after incubation for 30 min with 10 μM of 2-AG. As shown in Figure 3B,C, MH inhibited PGE_2_-GE formation with a higher potency (IC_50_ value: 301 (257 to 490) nM) compared to PGE_2_ (IC_50_ value: 1.21 (0.792 to 1.81) μM) in both systems. The mass chromatograms of PGE_2_ and PGE_2_-GE formed upon incubation of LPS-stimulated RAW264.7 cell homogenates as well as from intact cells incubated with 2-AG are shown in Figure 4. The LC-MS/MS method was validated by measuring the non-selective COX-2 inhibitor DuP-697 which showed the same potency in inhibiting the oxygenation of ECs and AA (Figure 3A,B,C,D). RAW264.7 cells can be stimulated to produce significant levels of 2-AG by raising intracellular levels of [Ca^2+^]. Cells were stimulated for 8 h with LPS, ATP, and thapsigargin in the presence of vehicle or different concentrations of MH. Figure 5A, B shows the concentration-dependent inhibition of PGE_2_ and PGE_2_-GE formation by MH. Intriguingly, already at low concentrations (10 to 30 nM), MH led to 20% to 25% inhibition of 2-AG oxygenation while AA oxygenation was not affected. At high concentrations (100 to 300 nM), MH inhibited the formation of both prostaglandin types but with more pronounced effects on PGE_2_-GE. At 1 μM, MH almost fully inhibited COX-2-mediated oxygenation of AA and 2-AG lacking the substrate specificity (Figure 5C). Since the absolute amount of PGE_2_ formed was about 100-times higher than PGE_2_-GE, we wanted to exclude a potential bias in the sensitivity of the system due to different amounts of the initial substrates (AA and 2-AG). Therefore, we tested the effects of DuP-697, and the results confirmed that this non-selective inhibitor potently blocked the formation of PGE_2_ and PGE_2_-GE with the same potency (Additional file 1: Figure S3E). To assess the relevance of COX-2 SSI *in vivo,* we evaluated the effects of MH in LPS-challenged Swiss albino mice. Animals were injected intraperitoneally (i.p.) with vehicle or different concentrations of MH and after 1 h, challenged for 6 h with LPS. Brain levels of AEA, 2-AG, AA, and PGE_2_, expressed as nmol (or pmol) mg^−1^ of tissue are shown in Figure 6A,B,C,D. Upon LPS injection, the amount of AA, 2-AG, and AEA did not significantly change, while PGE_2_ and corticosterone strongly increased (Figure 6E), as expected, and in agreement with the literature [34,35]. MH treatment did not affect the amount of AA and corticosterone at any concentration, while it slightly reduced the levels of PGE_2_ only at 20 mg kg^−1^. MH did not significantly affect AEA levels, while significantly increasing the amount of free 2-AG at 10 and 20 mg kg^−1^, with a tendency of increase also at the lowest dose tested (3 mg kg^−1^). Since 2-AG levels are about 500 to 1,000 times higher than AEA levels in Swiss albino mice, and assuming that both ECs are equipotent substrates for COX-2, it is not unexpected to see this more pronounced effect (doubling the amount) of a SSI COX-2 inhibitor on 2-AG levels. PGE_2_-EA and PGE_2_-GE were below the limit of detection. Moreover, NA-GABA (*N*-arachidonoyl GABA) and other *N*-acylethanolamines were not significantly affected by MH (Additional file 1: Figure S4). These findings suggest that MH maintained the ability to specifically inhibit COX-2-mediated EC oxygenation *in vivo* without affecting the oxygenation of AA. Several publications have recently demonstrated *in vitro* and *in vivo* beneficial effects of MH in neurodegenerative disease models [2,4,36]. Nonetheless, the penetration of the compound across the blood-brain barrier has never been investigated. We therefore established an LC-MS/MS method to quantify MH in the brain. Data show that after 7 h after the injection of 3, 10, and 20 mg kg^−1^, the brain contains 120.1 (93.6 to 147.5), 239.8 (121.1 to 342.3), and 350.2 (248.1 to 426.9) pmol g^−1^ of MH, respectively (Figure 6F). These results correlate with the significant increase of EC levels induced by 10 and 20 mg kg^−1^ of MH, while only showing a tendency at 3 mg kg^−1^. An increase of 2-AG levels can also be achieved by inhibiting its hydrolysis. Therefore, we tested the effects of MH on all the major EC hydrolytic enzymes (FAAH, MAGL, ABHDs) confirming that it does neither affect AEA nor 2-AG hydrolysis (Table 1), while the positive controls URB597, JZL184, and WWL70/tetrahydrolipstatin (for FAAH, MAGL, and ABHDs, respectively) inhibited EC hydrolysis as in agreement with the literature (data not shown) [22]. The trafficking of ECs across plasma membranes is likely regulated by a putative EC membrane transporter (EMT), and several inhibitors prevent intracellular accumulation of AEA and 2-AG [37]. MH only weakly inhibited the cellular accumulation of [^3^H]AEA in U937 cells with an IC_50_ value of 6.5 (4.7 to 9) μM (Table 1). A similar potency (EC_50_ value of 2.7 (1.1 to 6.5) μM) was observed using U937 cells differentiated into macrophages which lack the [^3^H]ethanolamine incorporation into phospholipids (as shown previously [37]), therefore ruling out potential effects on other targets indirectly related to the process of [^3^H]AEA uptake (Additional file 1: Figure S5). In the same system, the positive control UCM707 inhibited [^3^H]AEA uptake with an IC_50_ value of 1.6 (0.8 to 2.5) μM and 2.4 (1.2 to 3.6) μM in U937 cells and U937 cells differentiated into macrophages, respectively, in agreement with previous reports [23,37]. Thus, with nanomolar brain concentrations of MH, the inhibition of EC trafficking is unlikely to contribute to raising 2-AG levels *in vivo*.

**Table 1:** **Summary of the pharmacological effects exerted by MH, 27a, 32, and 34a**

**IC**_**50**_ **or** ***K***_**i**_ **values (mean value ± SD, μM)**												
	***h*** **COX-2**		**RAW264.7 Intact cells**		**CHO membranes**			**CHO cells**	**Pig brain homog.**		**BV-2 homog.**	**U937 cells**
	**AA**	**2-AG**	**AA**	**2-AG**	***h*** **CB**_**1**_	***h*** **CB**_**2**_	***h*** **CB2 [** ^**35**^ **S] GTPγS**	***h*** **CB2 cAMP**	**FAAH**	**MAGL**	**ABHDs**	**AEA uptake**
**MH**	0.58 ± 0.08	^**^0.15 ± 0.09	1.56 ± 0.12	^**^0.28 ± 0.09	2.4 ± 0.6	0.19 ± 0.12	0.20 ± 0.10	0.67 ± 0.07	>10	>10	>10	6.5 ± 1.2
**27a**	1.21 ± 0.08	^**^0.42 ± 0.09	0.98 ± 0.08	^**^0.28 ± 0.11	0.79 ± 0.10	0.11 ± 0.09	0.18 ± 0.09	0.71 ± 0.09	>10	>10	>10	>10
**32**	10.2 ± 0.12	^**^0.63 ± 0.05	2.8 ± 0.22	^**^0.21 ± 0.14	>10	>10	n.d.	n.d.	>10	>10	>10	6.3 ± 1.6
**34a**	4.31 ± 0.10	^*^1.29 ± 0.07	>30	^**^1.57 ± 0.13	1.12 ± 0.15	0.35 ± 0.11	1.82 ± 0.14	1.52 ± 0.13	>10	>10	>10	>10
**DuP-697**	0.061 ± 0.005	0.07 ± 0.01	0.004 ± 0.004	0.005 ± 0.006	n.d.	n.d.	n.d.	n.d.	n.d.	n.d.	n.d.	n.d.

The data are expressed as mean ± SD of at least three independent experiments each performed in triplicates. Binding affinity to CB_1_ and CB_2_ receptors is expressed as *K*_i_ value while modulation of CB_2_ receptor activation (cAMP and [^35^S] GTPγS) and inhibition of enzyme activity and AEA uptake are expressed as IC_50_ value. *P < 0.05; **P < 0.01 PGE_2_-GE *vs.* PGE_2_. n.d.: not determined *P* < 0.05; **P < 0.01 PGE_2_-GE *vs.* PGE_2_. Items in bold are compound abbreviations.

**Figure 7:**
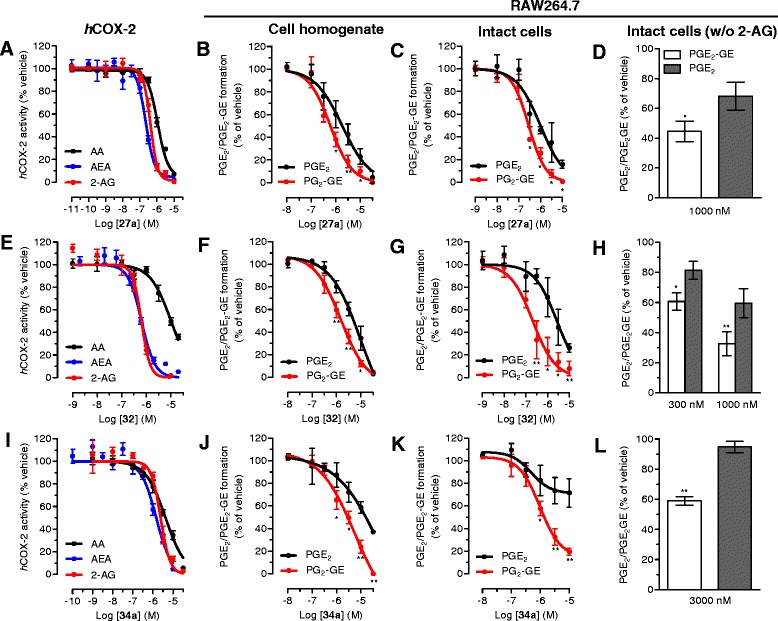
COX-2 inhibition by biphenyl neolignans in different biological matrices. (A-D) **27a**, (E-H) **32**, and (I-L) **34a** inhibition of 2-AG, AEA, and AA oxygenation in *h*COX-2 **(A, E, I)**, RAW264.7 cell homogenates **(B, F, J),** and intact cells **(C, G, K)** incubated with 10 μM of substrate (2-AG, AEA, or AA depending on the assay). **(D, H, L)** Inhibition of PGE_2_ and PGE_2_-GE formation in RAW264.7 cells upon LPS (1 mg mL^−1^) ATP (1 mM) and thapsigargin (2 μM) treatment without externally adding 2-AG. ^*^ *P* < 0.05, ^**^ *P* < 0.01 PGE_2_-GE *vs.* PGE_2_.

**Figure 8:**
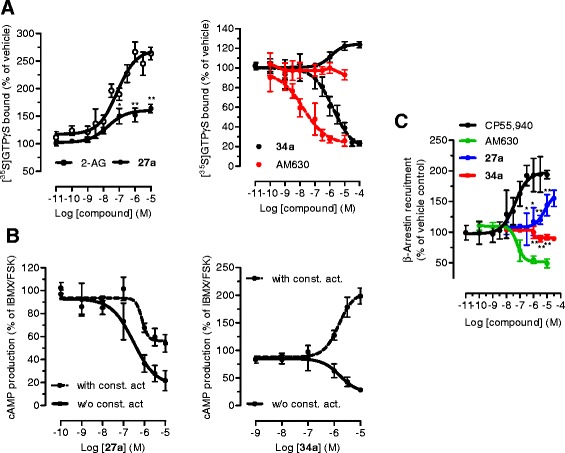
Modulation of CB_2_ receptor activity by **27a** and **34a. (A)** [^35^S]GTPγS binding assay performed in CHO-K1 membranes containing *h*CB_2_ receptors, in the presence of different concentrations of **27a** and **34a**. The CB_2_ inverse agonist AM630 is reported as positive control (red line). Experiments were performed in the presence (dotted line) and in the absence (solid line) of constitutive activity of the receptors. **(B)** Inhibition of forskolin-induced cAMP formation by **27a** and **34a** in CHO-*h*CB_2_ cells transfected with pGloSensorTM 22-F plasmid. The experiments were performed in the presence (dotted line) and in the absence (solid line) of constitutive activity. **(C)** *β*-arrestin recruitment induced by increasing concentrations of **27a** and **34a**, measured in PathHunter® *β*-arrestin cells (CHO-K1-HOMSA-CNR2). ^*^ *P* < 0.05, ^**^ *P* < 0.01 **27a** *vs.* 2-AG (Figure 8A); ^*^ *P* < 0.05, ^**^ *P* < 0.01 **27a** and **34a** *vs.* CP55, 940 (Figure 8C).

We tested a library of 40 MH, magnolol, and honokiol analogs for COX-2 SSI previously described [1,7]. Compounds which showed >50% inhibition of 2-AG oxygenation at 2 μM were selected for further investigations. As shown in Additional file 1: Figure S6A, B, from the whole library, only three molecules (**27a**, **32**, and **34a**, see Figure 1 for chemical structures) showed a stronger, selective inhibition of 2-AG oxygenation over AA. We characterized the effects of these three compounds in purified *h*COX-2, RAW264.7 cell homogenates, and intact cells similarly to MH. As shown in Figure 7, all compounds selectively inhibited 2-AG oxygenation with potencies ranging from 210 nM (**32**) to 1.57 μM in living cells (**34a**). Compounds **32** and **34a** showed the greatest selectivity for 2-AG oxygenation (10/15-fold more potent than AA oxygenation (Table 1)). At 10 μM, **32** did not show any relevant binding at both CB receptor subtypes, while **27a** and **34a** showed selective CB_2_ binding (Table 1). Next, we characterized the functional effects of both ligands by assessing G-protein and *β*-arrestin recruitment and cAMP formation. Compound **27a**, which was previously reported to be an inverse agonist [1], in our settings like MH behaved as a partial agonist in the [^35^S]GTPγS assay (Figure 8A), exhibiting full agonism at inhibiting the forskolin-induced cAMP production (Figure 8B) and sub-maximal recruitment of *β*-arrestin (Figure 8C). However, compound **34a** reduced [^35^S]GTPγS binding and increased cAMP formation, thus behaving as an inverse agonist (Figure 8A, B). Interestingly, when the constitutive activity was removed, **34a** behaved as an agonist by increasing the [^35^S]GTPγS binding and inhibiting the forskolin-induced cAMP formation (Figure 8A, B). This dual effect is a typical feature of ‘protean agonists’ [29]. In the *β*-arrestin assay, **34a** did not show any significant effect despite a tendency to weakly reduce *β*-arrestin recruitment by 10% (Figure 8C). The IC_50_ values of **27a** and **34a** for the [^35^S]GTPγS assay and cAMP assay are reported in Table 1. All three compounds (**27a**, **32**, and **34a**) did not show any significant interaction with other components of the ECS apart from the magnolol derivative (**32)** which weakly inhibited the [^3^H]AEA uptake (IC_50_ value of 6.3 (3.2 to 9.2) μM) similarly to MH. Similarly to MH, also compound **32** showed the same potency of inhibiting [^3^H]AEA uptake in U937 cells and U937-derived macrophages (EC_50_ value of 2.4 (0.9 to 6.1 μM) (Additional file 1: Figure S5).

## Discussion

MH is a selective and potent CB_2_ ligand which was originally described as a full agonist at intracellular [Ca^2+^] and an inverse agonist at Gi/o recruitment by Schuehly *et al.* [1]. Recently, another group reported the agonist behavior of MH also on the cAMP pathway [9]. In the present study, using different standardized receptor signaling assays, we found that MH behaves as a partial agonist in the [^35^S]GTPγS binding in overexpressing systems and native conditions and in *β*-arrestin recruitment assays, while acting as a full agonist at the cAMP pathway. Similar differential actions between the G-protein recruitment and adenylate cyclase modulation have been reported for other GPCRs like. For example, morphine and fentanyl induced total inhibition of cAMP formation upon binding to μ-opioid receptors, while only showing 75% to 80% of maximal G-protein activation compared to the full agonist etorphine [38]. The [^35^S]GTPγS binding assay monitors the first step of G-protein activation but does not provide further information about the downstream signaling pathways which can be differently affected by different ligands. Thus, GPCRs may be coupled prevalently with one type of G-protein, but depending on the system (native *vs.* overexpressing) and the type of tissue, the same receptor might activate different types of G-protein [28,30]. For example, CB_1_ receptors are usually coupled to Gi/o but under certain conditions can also recruit stimulatory G-protein [39,40], or can induce pertussis toxin-independent increase of intracellular [Ca^2+^] via Gq/11 recruitment [41]. It has been reported that CB_1_ receptors expressed in HEK-293 cells can transduce the signal via Gi/o when activated by CP55,940, 2-AG, or ∆^9^-THC, while WIN55,212-2 induced a CB_1_-mediated Gq/11 signaling [41]. This suggests that different ligands can trigger distinct active conformations of the receptor which, in turn, can recruit different types of G-proteins. In addition, ligands can also alternatively transduce the signal either via Gα- or Gβγ-subunit as it was recently proposed for ∆^9^-THC and 2-AG which modulate COX-2 expression in opposite ways by activating CB_1_ receptors in neurons [42]. The level of complexity is also raised by G-protein-independent signaling pathways such as *β*-arrestin, whose receptor-mediated recruitment can modulate several downstream effectors (e.g., MAPK, ERK1/2, PI3K) besides its role in the desensitization and internalization process (see [43] for review). The introduction of the ‘biased agonism’ (or functional selectivity) concept triggered a shift from the classic definition of agonism, antagonism, and inverse agonism towards the characterization of ligands which can preferentially activate only one (or few) signaling pathways [44]. It is well established that CB_2_ receptors possess high levels of constitutive activity and a high degree of trafficking from intracellular pools to the membrane surface and *vice versa* [45]. As reported by Schuehly *et al.* [1], MH and **27a** inhibit osteoclastogenesis by acting as inverse agonists on the CB_2_-mediated cAMP pathway. We now show that both ligands behave as full agonists by inhibiting cAMP formation in the presence or in the absence of constitutive CB_2_ activation. On the other hand, MH and **27a** show partial agonism for G-protein activation and *β-*arrestin recruitment. Partial agonists are very ductile pharmacological tools which can modulate the activation state of a certain receptor in both ways. Indeed, MH could either potentiate or attenuate the CB_2_-mediated G-protein recruitment triggered by residual 2-AG, depending on the concentration ratio between the two ligands. This pharmacological feature might at least in part explain the discrepancy between the different effects reported for MH at CB_2_ receptors (agonist *vs.* inverse agonist), due to the presence of high or low amounts of 2-AG in the culture medium. We recently reported that fetal bovine serum (FBS) contains ECs, and, depending on the batch, the levels of 2-AG can even reach sub-micromolar concentrations [46]. Culturing RAW264.7 cells with 2-AG high-content sera led to significant formation of osteoclasts [46]. In this scenario, the partial agonists MH and **27a** might exert their anti-osteoclastogenesis effects indirectly by blocking 2-AG-induced CB_2_-mediated osteoclastogenesis. Our current results also suggest that alternative signaling pathways than cAMP might be involved. In line with this hypothesis, Schuehly *et al.* [1] reported the agonistic behavior of MH on CB_2_-mediated increase of intracellular [Ca^2+^], while here, we report that MH triggers the recruitment of *β*-arrestin, which might contribute to the modulation of different downstream effectors. In addition, we described that the 2-*O*-butyl honokiol derivative (**34a**) acts as a ‘protean agonist’ as it could behave as an agonist or inverse agonist, depending on the proportion of constitutively activated CB_2_ receptors. On the contrary the CB_2_ inverse agonist AM630 switched from an inverse agonist to an inactive ligand upon the removal of the constitutive activity of the receptors in both [^35^S]GTPγS and cAMP assays (see Figure 8A and Additional file 1: Figure S1), thus confirming that **34a** is not simply behaving as an inverse agonist. Noteworthy, upon i.p. application, MH treatment significantly increased the brain levels of 2-AG in a concentration-dependent manner without significantly affecting AEA, AA, PGE_2,_ and corticosterone levels. These findings clearly suggest that the inhibition of COX-2-mediated oxygenation as metabolic pathway for EC degradation in conditions of inflammation is a relevant target for MH. Unfortunately, *in vivo* we and others could not detect PGE_2_-GE, probably due to an extensive hydrolysis to the related arachidonoyl derivative (PGE_2_), as previously shown [47,48]. Also, the effect of SSIs may turn out to be different, dependent on mouse strains. Recently, Hermanson *et al.* [21] reported using a morpholino amide derivative of indomethacin, LM-4131, a 150% increase of AEA levels and only minor effects on 2-AG levels (~110%) in imprinting control region (ICR) mouse brain. In normal conditions, 2-AG predominantly undergoes hydrolytic degradation [49,50], but upon LPS-challenge, COX-2 levels significantly rise in neurons [51] and astrocytes/microglial cells [52], thus increasing the relevance of the oxygenation pathway. In our setting we detected a significant increase of 2-AG levels by 170% and 200% upon treatment with 10 and 20 mg kg^−1^ of MH, respectively. AEA levels were not significantly changed by MH treatment despite a tendency to increase (120%) at the dose of 20 mg kg^−1^. In addition, MH treatment selectively modulated the levels of 2-AG without affecting the levels of AA, PGE_2_, the FAAH-substrates *N*-acetylethanolamines, and corticosterone. Therefore, SSI of COX-2 activity appears to be a promising strategy to modulate EC levels in the brain, especially under inflammatory conditions. Prostamides and PG-GEs play an active role in the inflammatory process both *in vitro* and *in vivo* by acting through CB_1/2_ receptor-independent targets [15,19,53]. In hippocampal neurons, COX-2 inhibition rather than FAAH blockage led to a prolonged depolarization-induced suppression of inhibition suggesting a prominent role of 2-AG in the retrograde signal and COX-2-mediated oxygenation as relevant mechanism of reducing 2-AG levels at the synaptic level [54]. Straiker *et al.* [55] also reported the relevant role of COX-2 activity in reducing EC-mediated retrograde signaling. Recently it was shown that MH can weakly potentiate GABA-induced chloride currents in *Xenopus* oocytes by acting at the most common α_1_β_2_γ_2_ GABA_A_ receptor subtype [8]. In an animal model, repeated low doses (0.5 mg kg^−1^*per os*) of MH produced weak anxiolytic effects [56] similar to the SSI LM-4131 [21]. Thus, MH could represent a prototype of multi-target compound which can directly and indirectly potentiate the ECS. Nonetheless, the pharmacokinetic profile of MH indicates a poor oral bioavailability and extensive hepatic metabolism in rats [57]. Our data show that upon i.p. injection, MH penetrates the brain at concentrations which are in line with the effects on CB_2_ receptors and the SSI of COX-2 activity (approximately 200 and 300 nM for 10 and 20 mg kg^−1^, respectively). Mice were sacrificed 7 h after the injection, thus indicating that MH could reach and accumulate in the brain without being significantly metabolized.

Despite the reported poor bioavailability after single oral administration [57], upon repeated doses, MH might accumulate and exert pharmacological effects in the brain. This could also explain the beneficial effects reported for long-term treatment with low doses of MH administered *per os* (0.1 to 1 mg kg^−1^) in neurodegenerative animal models [2,4,36]. CB_2_ receptors are almost absent in the CNS under normal conditions, but upon inflammation, they are significantly upregulated in microglial cells. Neuroinflammation is a hallmark of neurodegenerative diseases which contributes to the onset and progression of the disease [58,59] and several results support the positive role of CB_2_ activation in neurodegenerative diseases including the stimulation of *β*-amyloid removal by macrophages and down-regulation of TNF-α production [60-62]. MH showed beneficial effects in Alzheimer’s disease (AD) models by reducing LPS-induced *β*-amyloid accumulation, pro-inflammatory cytokine production, and memory impairment [2,4]. Since in addition to the direct activation of CB_2_ receptors, MH also elevates 2-AG levels, which is the main endogenous agonist for CB_2_, and it further inhibits the formation of pro-inflammatory PG-EAs and PG-GEs; synergistic effects for attenuating the inflammatory process are expected to represent one of the main underlying mechanisms of the neuroprotective effects exerted by MH. Interestingly, (*R*)-flurbiprofen is a COX-2 SSI with negligible effects on AA metabolism [14] which showed COX-2-independent anti-inflammatory effects in AD and experimental autoimmune encephalomyelitis multiple sclerosis models [63-65]. Remarkably, in this model, (*R*)-flurbiprofen induced a significant increase of EC levels in the spinal cord and somatosensory cortex while only mildly affecting PGs levels [65]. Therefore, based on our data we suggest that the major mode of action related to the pronounced neuroprotective effects of MH may be related to its outstanding polypharmacology in the ECS.

## Conclusions

We show for the first time that MH penetrates the blood-brain barrier and accumulates in the brain in amounts that are consistent with the concentrations that modulate the ECS *in vitro*. MH might serve as prototype scaffold to develop compounds exhibiting a novel combination of polypharmacology to modulate the ECS through a concurrent activation of CB_2_ receptors and the SSI of COX-2-mediated EC oxygenation. For MH, the latter mechanism was shown here for the first time both *in vitro* and *in vivo*. This polypharmacology might achieve synergistic anti-inflammatory and protective actions in certain tissues, including the brain, by directly and indirectly ( increasing 2-AG levels) activating CB_2_ receptors, avoiding the typical gastrointestinal and cardiovascular side effects of NSAIDs. Moreover, these two mechanisms of action might be more relevant in neuroinflammatory diseases where both COX-2 and CB_2_ receptors are overexpressed in the brain, thus providing a rationale for the diverse neuroprotective effects reported for MH in animal models.

## Additional file

Additional file 1: Figure S1. [35S] GTPS binding assays performed in mock-transfected CHO cells. **Figure S2:** Pharmacological behavior of AM630 and CP55,940 in the presence or absence of constitutive activity of CB2 receptors. **Figure S3:** The effects of DuP-697 in different cellular systems. **Figure S4:** LC-MS/MS quantification of *N*-acetylethanolamines in mouse brain. **Figure S5:** [3H] AEA uptake into U937 cell-derived macrophages. **Figure S6:** Screening of several MHK, honokiol, and magnolol derivatives for SSI of COX-2 activity.

## Abbreviations

AA: arachidonic acid
AD: Alzheimer’s disease
ABHDs: α, β-hydrolases
AEA: anandamide
2-AG: 2-arachidonoyl glycerol
AM630: [6-iodo-2-methyl-1-[2-(4-morpholinyl) ethyl]-1H-indol-3-yl] (4-methoxyphenyl)-methanone
cAMP: cyclic AMP
BSA: bovine serum albumin
CB_1_: type-1 cannabinoid receptor
CB_2_: type-2 cannabinoid receptor
CP55: 940, (−)-cis-3-[2-hydroxy-4-(1,1 dimethylheptyl) phenyl]-trans-4-(3-hydroxypropyl) cyclohexanol
COX-2: cyclooxygenase-2
CNS: central nervous system
EC: endocannabinoid
ECS: endocannabinoid system
FAAH: fatty acid amide hydrolase
FSK: forskolin
ICR: imprinting control region
IS: internal standard
JZL184: 4-nitrophenyl-4-[bis (1,3-benzodioxol-5-yl) (hydroxy) methyl] piperidine-1-carboxylate
MAGL: monoacylglycerol lipase
MH: 4′-*O*-methylhonokiol
MRM: multiple reaction monitoring
NA-GABA: *N*-arachidonoyl GABA
NSAIDs: non-steroidal anti-inflammatory drugs
PGs: prostaglandins
PG-EAs: prostaglandin-ethanolamides (prostamides)
PG-GEs: prostaglandin glycerol esters
SSI: substrate selective inhibition/inhibitor
UCM707: (5Z, 8Z, 11Z, 14Z)-N-(3-furanylmethyl)-5,8,11,14-eicosatetraenamide
URB597: (3′-(aminocarbonyl) [1, 1′-biphenyl]-3-yl)-cyclohexylcarbamate
WIN55212-2: (R)-(+)-[2, 3-dihydro-5-methyl-3-(4-morpholinylmethyl) pyrrolo-[1,2,3-de]-1,4-benzoxazin-6-yl]-1-naphtaleneylmethanone
WWL70: N-methyl-N-[[3-(4-pyridinyl) phenyl] methyl]-carbamic acid 4′ - (aminocarbonyl) [1, 1′-biphenyl]-4-yl ester
