# Supplementary material for: 4′-O-methylhonokiol increases levels of 2-arachidonoyl glycerol in mouse brain via selective inhibition of its COX-2-mediated oxygenation
Source: J Neuroinflammation. 2015 May 13;12:89. doi: 10.1186/s12974-015-0307-7 (PMC4490613; doi:10.1186/s12974-015-0307-7)
Supplement: Additional file 1: Figure S1. — [35S] GTPS binding assays performed in mock-transfected CHO cells. Figure S2: Pharmacological behavior of AM630 and CP55,940 in the presence or absence of constitutive activity of CB2 receptors. Figure S3: The effects of DuP-697 in different cellular systems. Figure S4: LC-MS/MS quantification of N-acetylethanolamines in mouse brain. Figure S5: [3H] AEA uptake into U937 cell-derived macrophages. Figure S6: Screening of several MHK, honokiol, and magnolol derivatives for SSI of COX-2 activity. [file 12974_2015_307_MOESM1_ESM.pptx]

## Slide 1
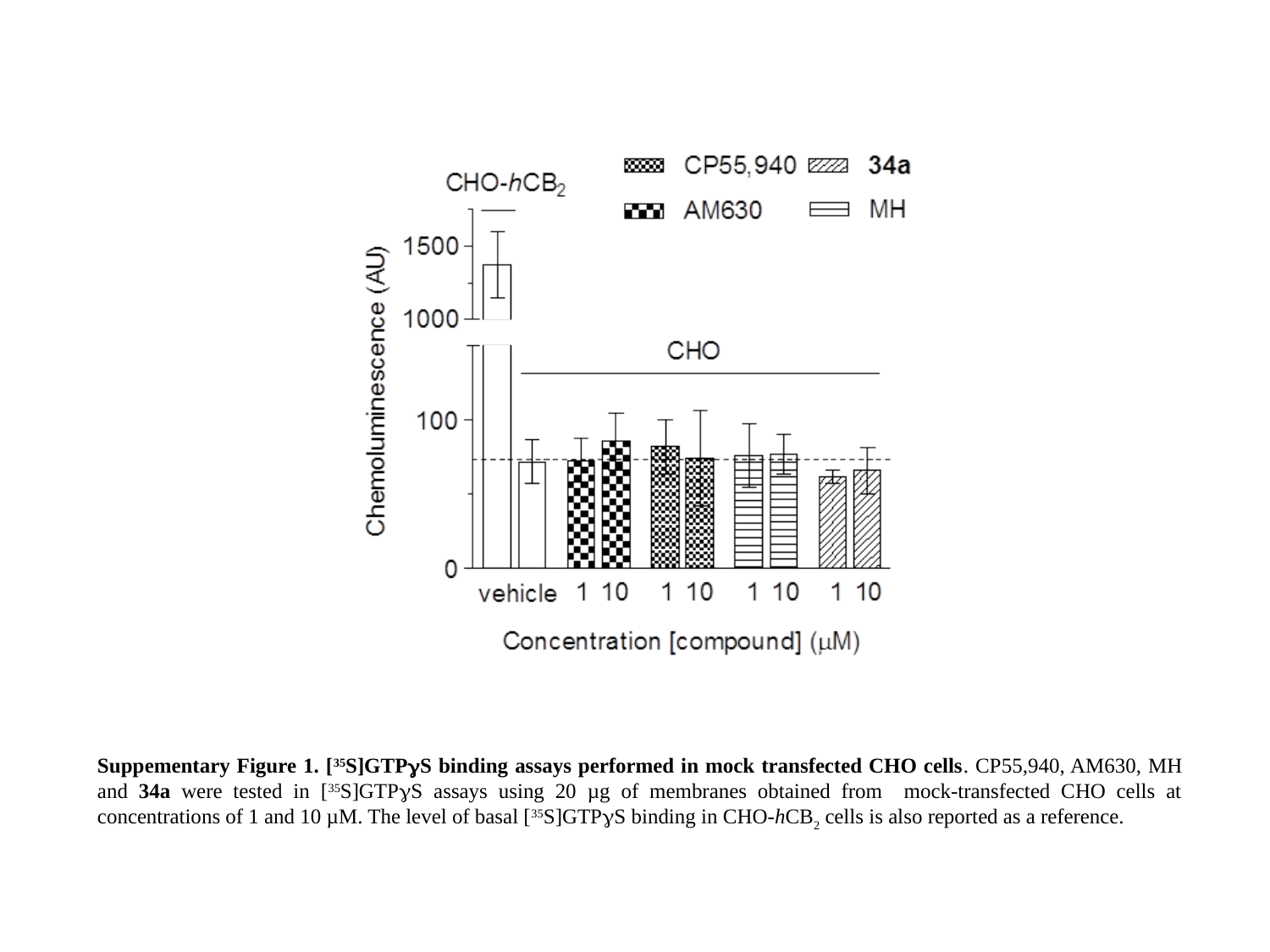

Suppementary Figure 1. [35S]GTPγS binding assays performed in mock transfected CHO cells. CP55,940, AM630, MH and 34a were tested in [35S]GTPγS assays using 20 µg of membranes obtained from mock-transfected CHO cells at concentrations of 1 and 10 µM. The level of basal [35S]GTPγS binding in CHO-hCB2 cells is also reported as a reference.

## Slide 2
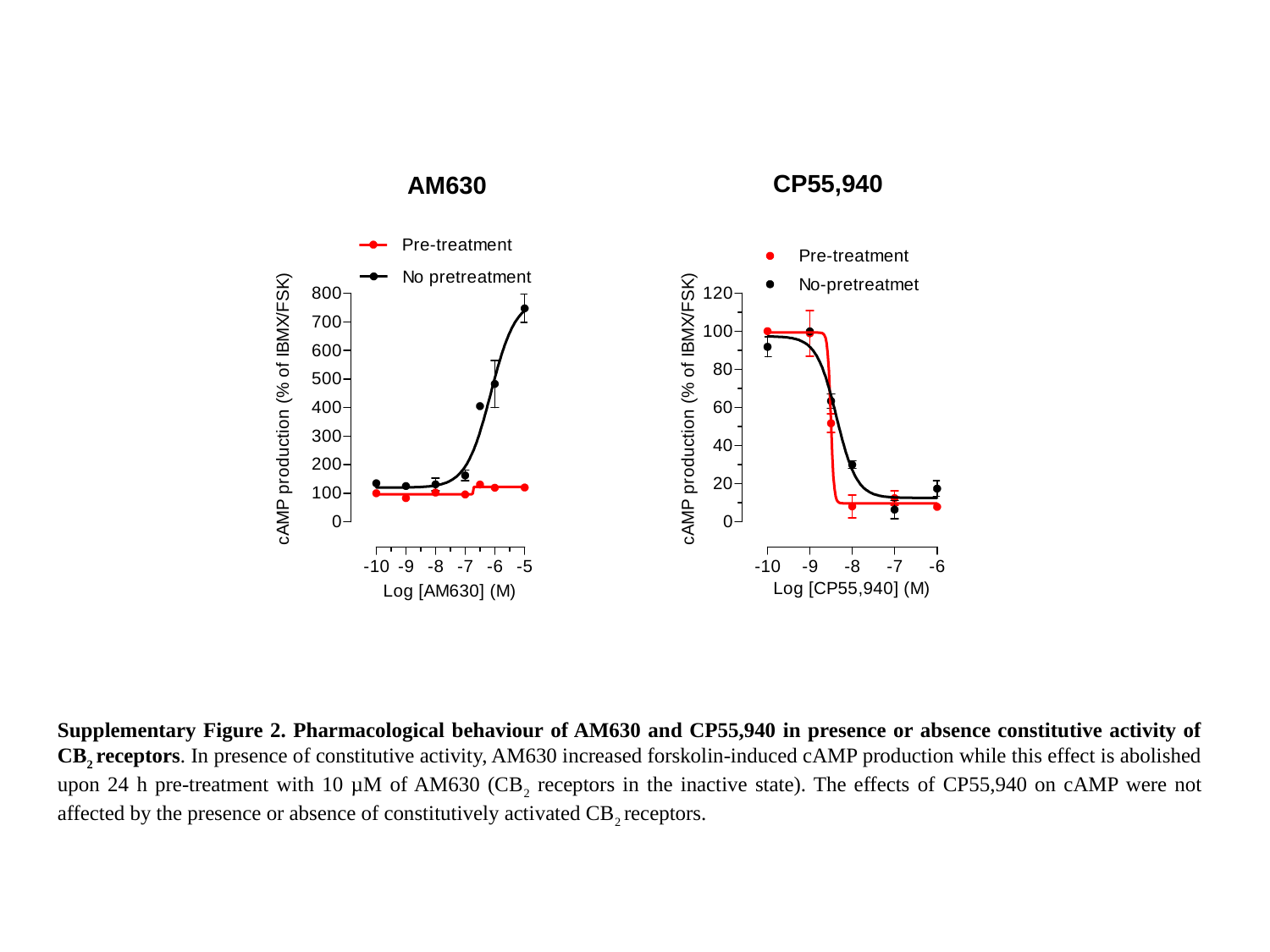

CP55,940
AM630
Supplementary Figure 2. Pharmacological behaviour of AM630 and CP55,940 in presence or absence constitutive activity of CB2 receptors. In presence of constitutive activity, AM630 increased forskolin-induced cAMP production while this effect is abolished upon 24 h pre-treatment with 10 µM of AM630 (CB2 receptors in the inactive state). The effects of CP55,940 on cAMP were not affected by the presence or absence of constitutively activated CB2 receptors.

## Slide 3
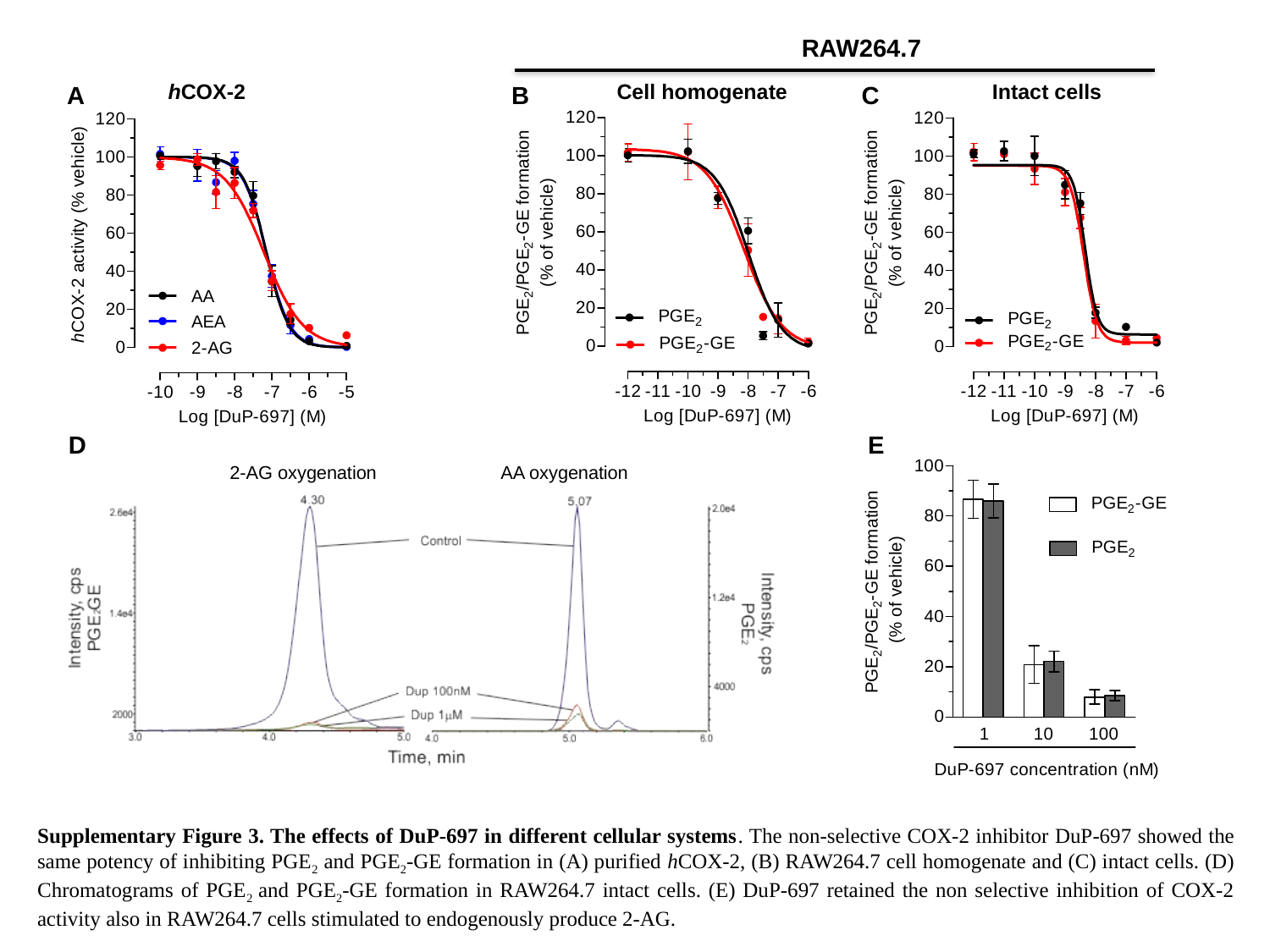

RAW264.7
Intact cells
hCOX-2
Cell homogenate
A
B
C
D
E
2-AG oxygenation
AA oxygenation
Supplementary Figure 3. The effects of DuP-697 in different cellular systems. The non-selective COX-2 inhibitor DuP-697 showed the same potency of inhibiting PGE2 and PGE2-GE formation in (A) purified hCOX-2, (B) RAW264.7 cell homogenate and (C) intact cells. (D) Chromatograms of PGE2 and PGE2-GE formation in RAW264.7 intact cells. (E) DuP-697 retained the non selective inhibition of COX-2 activity also in RAW264.7 cells stimulated to endogenously produce 2-AG.

## Slide 4
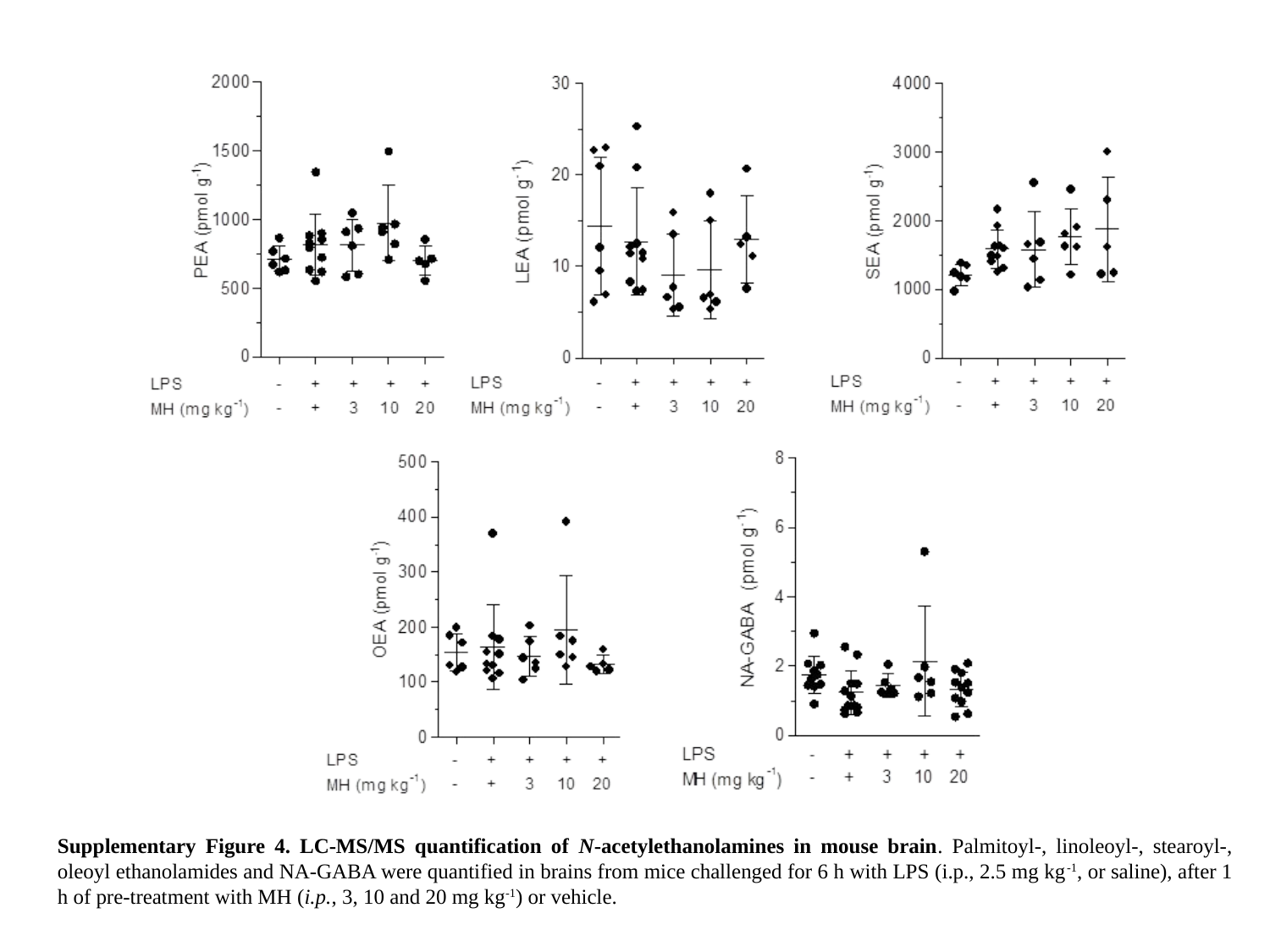

Supplementary Figure 4. LC-MS/MS quantification of N-acetylethanolamines in mouse brain. Palmitoyl-, linoleoyl-, stearoyl-, oleoyl ethanolamides and NA-GABA were quantified in brains from mice challenged for 6 h with LPS (i.p., 2.5 mg kg-1, or saline), after 1 h of pre-treatment with MH (i.p., 3, 10 and 20 mg kg-1) or vehicle.

## Slide 5
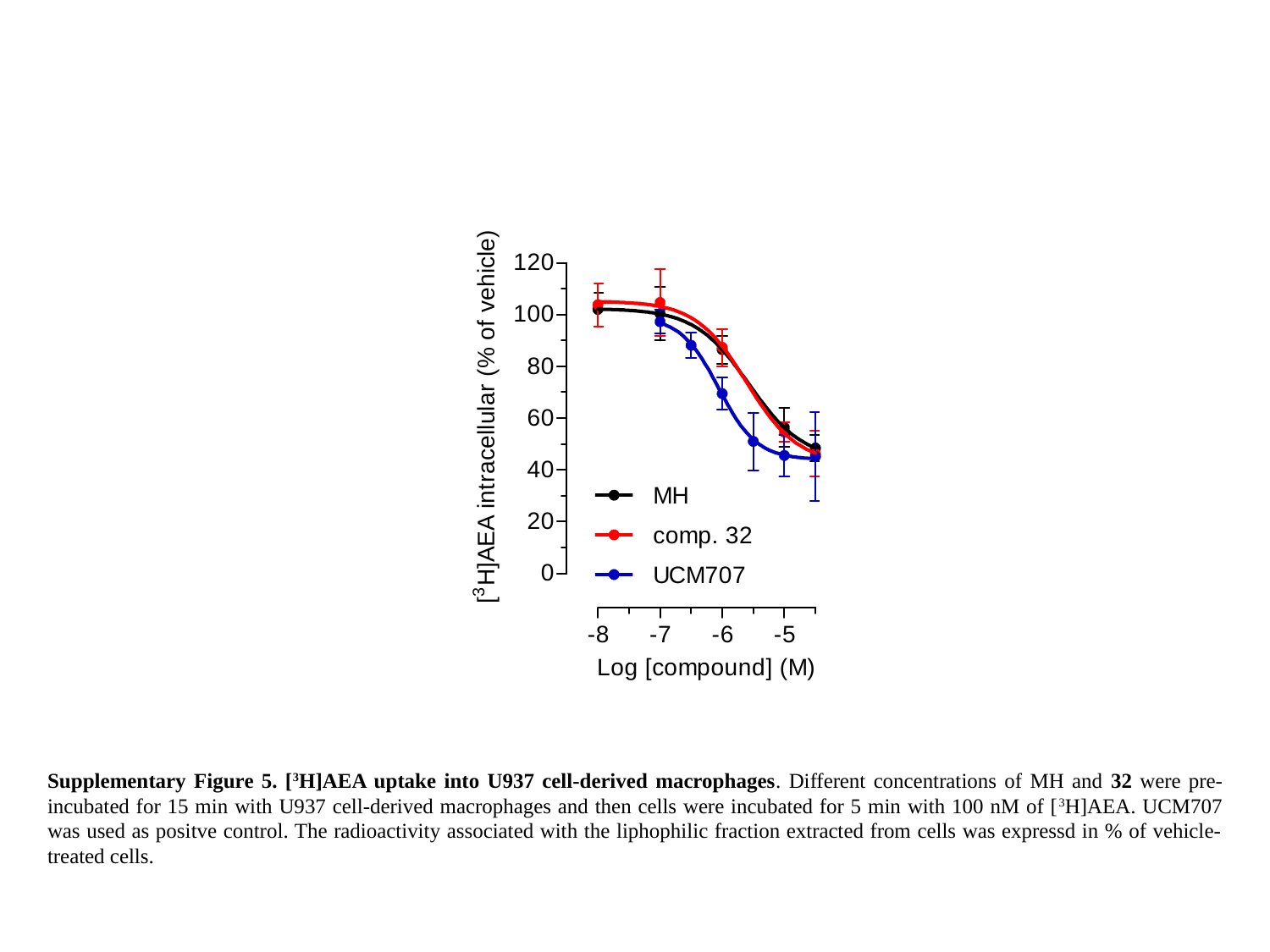

Supplementary Figure 5. [3H]AEA uptake into U937 cell-derived macrophages. Different concentrations of MH and 32 were pre-incubated for 15 min with U937 cell-derived macrophages and then cells were incubated for 5 min with 100 nM of [3H]AEA. UCM707 was used as positve control. The radioactivity associated with the liphophilic fraction extracted from cells was expressd in % of vehicle-treated cells.

## Slide 6
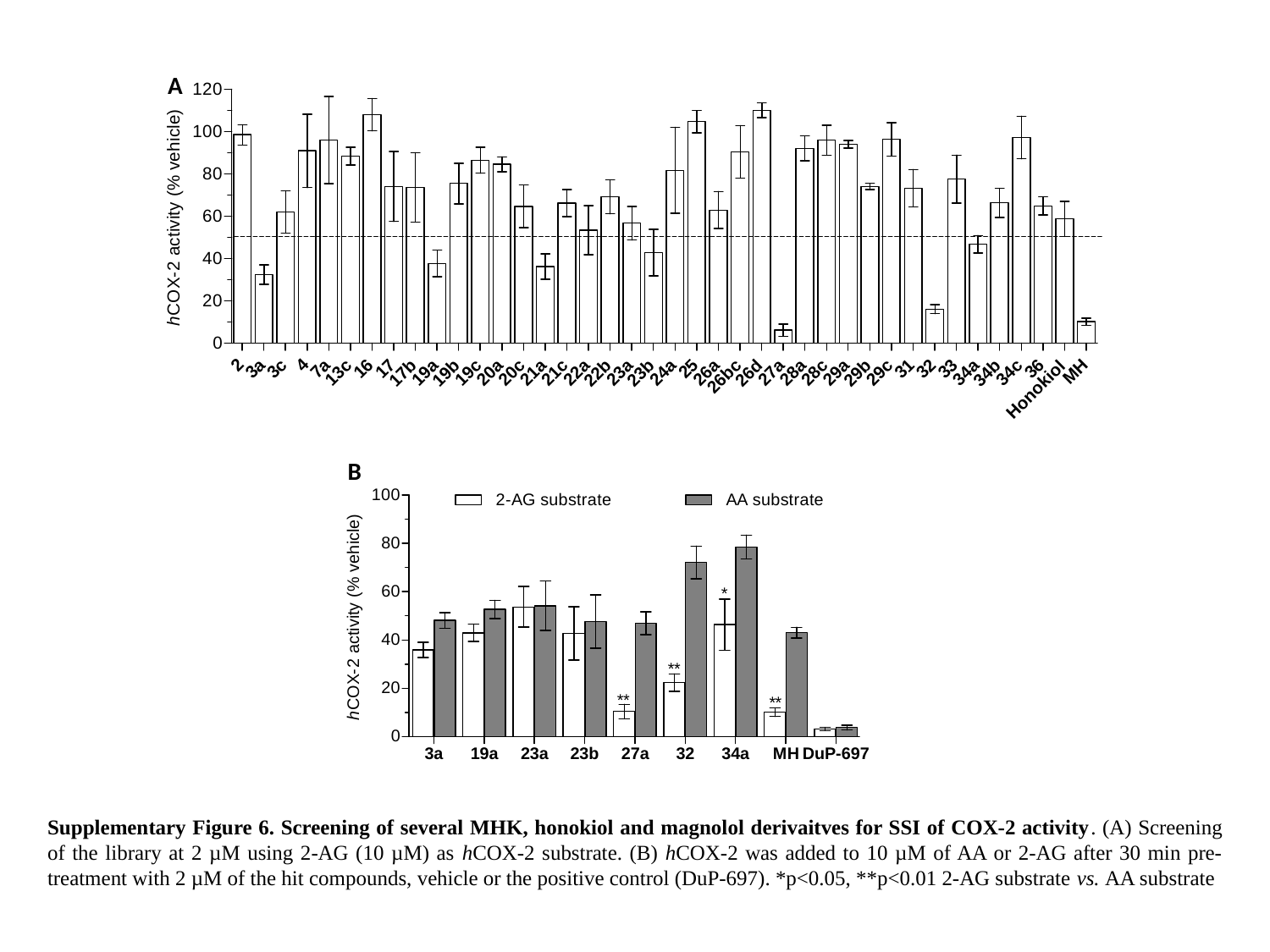

A
B
Supplementary Figure 6. Screening of several MHK, honokiol and magnolol derivaitves for SSI of COX-2 activity. (A) Screening of the library at 2 µM using 2-AG (10 µM) as hCOX-2 substrate. (B) hCOX-2 was added to 10 µM of AA or 2-AG after 30 min pre-treatment with 2 µM of the hit compounds, vehicle or the positive control (DuP-697). *p<0.05, **p<0.01 2-AG substrate vs. AA substrate
